# Efficacy of interventions to treat and prevent Alzheimer's disease in individuals with Down syndrome: A meta‐analysis and systematic review

**DOI:** 10.1002/alz70858_102403

**Published:** 2025-12-25

**Authors:** Emily E Munn, Anna Montelongo, Viraj K Patel, Jill C Fodstad, Mary Ciccarelli, Melissa M Pangelinan

**Affiliations:** ^1^ Indiana University Bloomington, Bloomington, IN, USA; ^2^ Indiana University School of Medicine, Bloomington, IN, USA; ^3^ Indiana University School of Medicine, Indianapolis, IN, USA

## Abstract

**Background:**

Down syndrome (DS) is a leading genetic risk factor for Alzheimer's disease (AD). The incidence of clinical symptoms of AD increases dramatically from 8% to 80% between the ages of 40‐54 years in persons with DS. The prevention and treatment for AD are well‐documented in persons without DS. However, less is known about prevention and treatment for those with DS. The purpose of this systematic review and meta‐analysis is to evaluate the efficacy of interventions for the prevention and treatment of AD in persons with DS.

**Method:**

The review was limited to articles with a pharmacological or non‐pharmocological program, intervention, or treatment. Studies were included if they were in English, peer‐reviewed, included adults ages 18‐69 diagnosed with Down syndrome, and published as of Dec 1^st^, 2023. Additionally, as this project focused on AD, only articles with dependent variables related to AD, dementia, or AD‐related cognitive and behavioral outcomes were included.

**Result:**

A total of 781 records were screened for inclusion. 107 abstracts were then included in a full‐text review. 25 articles with 1,417 individuals with DS ages 18‐69 met inclusion. Four types of interventions were examined: pharmacological (*n* = 18), exercise (*n* = 5), environmental (*n* = 1), and cognitive training (*n* = 1). A meta‐analysis was used to assess treatment effects on cognitive and behavioral outcomes. Overall, the interventions and treatments significantly improved cognitive and behavior outcomes (*t* (66) = 4.67, *p* <0.0001, *d* =0.29, 95% *CI*: 0.16‐0.40). However, there was high heterogeneity among these effects (*I^2^ = 93.8%*, 95%*CI*:92.8‐94.7).

**Conclusion:**

AD interventions appear effective for individuals with DS. Pharmacological interventions, specifically donepezil, anti‐depressants, EGCG, simvastatin, and nicotine, can improve a broad spectrum of AD outcomes. Exercise and cognitive training are also promising to reduce AD symptoms on this population.

